# Non-compliance to antiretroviral therapy readjustments following complications in HIV-positive patients in South Africa

**DOI:** 10.4102/jphia.v16i1.725

**Published:** 2025-04-30

**Authors:** Ntandoyakhe N. Nxumalo, Solomon Thule, Selente Bezuidenhout, Robert Summers, Elmien Bronkhorst

**Affiliations:** 1 Department of Clinical Pharmacy, School of Pharmacy, Sefako Makgatho Health Sciences University, Pretoria, South Africa; 2 College of Economic and Management Sciences, School of Management Sciences, University of South Africa, Pretoria, South Africa; 3 Department of Public Health Pharmacy and Management, School of Pharmacy, Sefako Makgatho Health Sciences University, Pretoria, South Africa; 4 School of Pharmacy, Sefako Makgatho Health Sciences University, Pretoria, South Africa

**Keywords:** antiretroviral stewardship, hepatic impairment, regimen adjustment, renal impairment, virological failure

## Abstract

**Background:**

South Africa accounts for 19% of the global population living with human immunodeficiency virus (HIV), limited knowledge exists on adherence to guidelines when managing complications.

**Aim:**

This study assesses regimen adjustment for HIV-positive patients with renal and hepatic dysfunction resulting from antiretroviral therapy (ART) according to the South African Treatment Guidelines and examines the re-initiation of ART in patients who have defaulted.

**Setting:**

The study was conducted at Tshepang HIV Clinic, Ga-Rankuwa, Pretoria, South Africa.

**Methods:**

A retrospective review was conducted between November 2020 and December 2020. Patients who had been on ART for over 18 months and presented with hepatic, renal and/or virological failure were included in the study. The data collection tool included demographics and medical records. Statistical Package for Social Sciences version 25 for Windows was used for data analysis.

**Results:**

A total of 181 files were reviewed, and only 37 were eligible for participation. The study found that treatment received by 25% of hepatic failure patients and 41.3% of renal impairment patients complied with South African ART guidelines. Furthermore, 40% of patients with virological failure were re-initiated according to guidelines.

**Conclusion:**

This study found practices that were contrary to the prescribed guidelines with non-compliance accounting for more than 40%.

**Contribution:**

This study demonstrates a high incidence of adherence failure to South African ART guidelines. Defaulted patients are placed risk of antiretroviral resistance. Adherence to guidelines is important to prevent complications resulting from ART.

## Introduction

Human immunodeficiency virus (HIV) is a chronic and incurable infectious disease characterised by a decline in the number of clusters of differentiation 4 (CD4) cells, which underlies the immunosuppression observed in affected individuals. When used optimally, antiretroviral therapy (ART) effectively reduces the risk of transmission and improves clinical outcomes and quality of life in people living with HIV.^1^

Patients initiated on Highly Active Antiretroviral Therapy (HAART) remain on medication indefinitely. A modification in the HAART regimen may become necessary because of possible acute or chronic toxicities, development of virological failure, defined as confirmed failure to suppress the viral load to less than 200 copies/mL, or the event of adverse drug events.^2^ Using the 2023 National Treatment Guidelines, individual ART drugs may be substituted in the event of toxicities and stewardship programmes of the National Department of Health of South Africa as guidance for the appropriate regimen adjustments.^3^

Nephrotoxicity, which is a poisonous effect of ART, such as tenofovir (TDF), requires close monitoring to ensure reversal during acute stages.^4^ While research has established that various mechanisms by which people living with HIV can develop liver injury, appropriate management of such patients remains a challenge.^5^

As a result of non-adherence to ART, patients are susceptible to medicine resistance. This emphasises the role of healthcare professionals in improving patient-centred outcomes and the prevention of virological failure in people living with HIV.

This study aimed to assess adherence to South African guidelines for ART regimen adjustments in patients with virological failure and those who developed hepatic and renal toxicities during their treatment. One principle of antiretroviral (ARV) stewardship aims to monitor drug-related errors and injuries and to correct them timeously. Antiretroviral stewardship interventions, such as rectifying incorrect regimens, dosages and drug interactions, can reduce adverse events and medication errors.^3^

## Research methods and design

### Study population and sampling strategy

The study was conducted at Tshepang Clinic, which is an HIV clinic located in Ga-Rankuwa, Pretoria, South Africa. The clinic attends to approximately 30 patients per day. Clinical markers, including weight and CD4 count, were recorded. The study was conducted over a period of 6 weeks.

Of the 181 patient records, 37 patient files met the inclusion criteria and were selected for the study. Patients with confirmed renal, hepatic or virological failure were included, provided they had been on ART for at least 18 months. The study followed a retrospective descriptive quantitative approach.

### Data collection

Patient records were reviewed to assess whether or not patients’ ART regimens were adjusted in line with the South African national consolidated guidelines when patients presented with hepatic, renal or virological failure.

The data collection occurred over a period of 6 weeks. Retrospective data from 18 months were reviewed in order to analyse at least three sets of blood results for each patient, which were sufficient to identify variations in patients’ virological, hepatic and renal function deviations.^6^

The data collection instrument was a self-developed tool that aimed to meet the objectives and aims of the study. Certain components of the tool were adapted from a study by Kaboli.^7^ It was validated in a pilot study before the commencement of the study within the facility, and the data collected for this purpose were excluded from the research study.

All necessary data including demographics, diagnosis and monitoring parameters such as viral load, CD4 count, urea, creatinine, estimated glomerular filtration rate and other concomitantly administered medicines were collected by reviewing the patient files in the clinic. This process identified patterns in regimen changes for patients who needed re-initiation after defaulting on ART and experiencing hepatic and/or renal function decline. Assessment of regimen adjustments in the presence of renal and/or hepatic disease, and defaulting ARV treatment, was carried out according to the HIV consolidated guidelines.^8^

### Data analysis

A retrospective descriptive analysis was conducted. Quantitative variables were summarised with means, medians, interquartile ranges (IQR) and percentages. Data were captured on a Microsoft Excel^™^ spreadsheet, imported to Statistical Package for Social Sciences (SPSS) version 25 and analysed descriptively using statistical models and descriptors. A 95% confidence interval was used to test statistical significance. Regimen adjustment patterns were summarised as percentages. The description of regimen adjustments was grouped as compliant and non-compliant under the categories of hepatic failure, renal failure and virological failure.

### Ethical considerations

Ethical clearance to conduct this study was obtained from the Sefako Makgatho Health Sciences University’s Research Ethics Committee (SMUREC) (reference no.: SMUREC/P/42/2020). Anonymity was maintained at all times by ensuring that the details of the patients remained confidential. All data were stored on a password-protected computer and only accessible by the researcher.

## Results

### Patient file selection and identification

**FIGURE 1:**
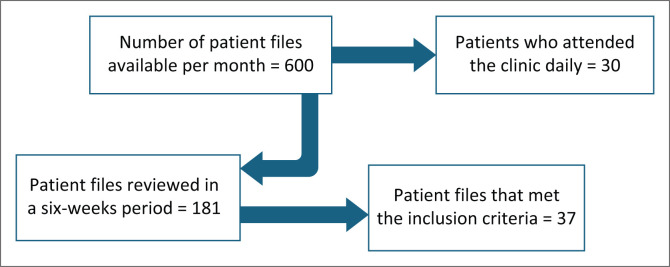
Identification of study population.

An overview of the selection process, which identified the study population from the facility of interest, is depicted in Figure 1.

### Demographic data

Table 1 describes the demographic information and clinical markers of patients who were included in the study (*n* = 37). The majority of study participants were female (*n* = 20; 54.05%) with a median age of 50 years (± standard deviation [s.d.]: 12.66).

**TABLE 1:** Demographic data and clinical markers.

Demographic and information and clinical markers	Results		Median	± s.d.	Mean	Average IQR	IQR	Ref range (500–1200 cells/mm^3^)
	*n*	%						
Patient age (years)	37	-	50	12.66	49.86	-	-	-
**Sex**	37	-	-	-	-	-	-	-
Female	20	54.05	-	-	-	-	-	-
Male	17	45.95	-	-	-	-	-	-
**Weight (six-monthly intervals)**								
Weight 1 (kg)	-	-	-	-	-	69.50	62.97–79.85	-
Weight 2 (kg)	-	-	-	-	-	71.80	67.99–84.95	-
Weight 3 (kg)	-	-	-	-	-	73.40	65.82–85.40	-
**CD4 count (six-monthly intervals)**								
CD4 1 (cells/mm^3^)	-	-	-	-	-	-	400	238–602
CD4 2 (cells/mm^3^)	-	-	-	-	-	-	329	230–521
CD4 3 (cells/mm^3^)	-	-	-	-	-	-	269	201–507

IQR, interquartile range; kg, kilograms; CD4, cluster of differentiation 4.

Patients’ weight and CD4 counts were recorded at a 6-monthly interval, according to the 2023 Guidelines of the National Department of Health of South Africa. However, not all patient files had these data available. The first weight (Weight 1) was recorded for 34 (91.9%) patients, whereas only 33 (89.9%) files showed recorded weight at 12 months (Weight 2). Similarly, only 17 (45.9%) patient files included a record of CD4 counts at 18 months.

### Patients presenting with renal failure, hepatic failure and defaulting on antiretroviral treatment

**FIGURE 2:**
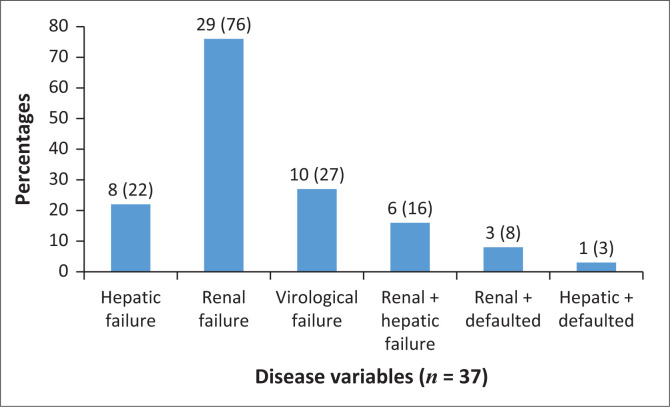
Percentages of patients with different disease variables.

Figure 2 describes the number of patients identified in different disease variables. Among the 37 patients, 29 (76%) presented with renal failure, 8 patients (*n* = 8; 22%) with hepatic failure and 10 patients defaulted from ART and presented with confirmed virological failure. Some patients presented with more than one disease variable, causing the variables to be higher than the number of patients; for example, six patients presented with both renal and hepatic failure (and three with virological and renal failure).

### Adjusted drug regimens – Adherence to guidelines

Table 2 presents the current treatment regimens prescribed and their compliance profile to the South African Treatment Guidelines. It depicts the number of patients with renal and hepatic failure, together with those who defaulted and presented with virological failure. Furthermore, it describes the drug regimens that participating patients were treated with. Seven patients with renal failure (*n* = 7; 24%) were non-compliant with guidelines and prescribed efavirenz (EFV) 600 mg, abacavir (ABC) 600 mg and lamivudine (3TC) 100 mg. Six patients (20.7%) complied with ART guidelines when adjusting regimens for patients who experienced renal failure, with their new regimen being EFV 600 mg, ABC 600 mg and 3TC 300 mg. Patients with renal failure with regimens that complied were recorded at 29 (41.3%). Furthermore, the overall compliance recorded for hepatic failure was 8 (25%) and 10 patients defaulted (25%).

**TABLE 2:** Adjusted drug regimens – Adherence to guidelines.

Indication	Compliance to guidelines (%)	Compliant regimen(s)	Non-compliance to guidelines (%)	Non-compliant regimen(s)
Renal failure	41.37	EFV 600+ ABC 600+ 3TC 300 (6)	58.62	EFV 600+ ABC 600+ 3TC 100 (7)
		AZT 600+ 3TC 300+ EFV 600 (3)		EFV 600+ ABC 600+ 3TC 500 (2)
		EFV 600+ ABC 600+ 3TC 150 (1)		EFV 600+ TDF 300 +FTC 200 (1)
		EFV 600+ ABC 300+ 3TC 150 (1)		AZT 600+ LPV/r 400/100 (1)
		LPV/r 800/200+ 3TC 300+ ABC 600 (1)		LPV/r 800/200+ 3TC 100+ ABC 600 (3)
				d4T 40+ 3TC 50+ EFV 600 (1)
				d4T 40+ 3TC 300+ EFV 600 (1)
				d4T 40+ 3TC 100+ EFV 600 (1)
Hepatic failure	25	TDF 300 + 3TC 300+ LPV/r 800/200 (2)	75	LPV/r 800/200+ 3TC 100+ ABC 600 (2)
				EFV 600+ 3TC 100+ ABC 600 (2)
				EFV 600+ 3TC 500+ ABC 600 (2)
Virological failure	40	AZT 600+ 3TC 300+ LPV/r 800/200 (2)	60	AZT 600+ 3TC 300+ EFV 600 (3)
		TDF 300 + 3TC 300+ LPV/r 800/200 (2)		EFV 600+ FTC 200+ TDF 300 (2)
				AZT 600+ LPV/r 400/100 (1)

Note: Measured according to the National Department of Health Treatment Guidelines, 2020.

3TC, lamivudine; ABC, abacavir; AZT, zidovudine; EFV, efavirenz; TDF, tenofovir; LPV/r, lopinavir/ritonavir; d4T, stavudine; FTC, emtricitabine.

The review of patient files revealed that some patients were taking additional medication for co-morbidities. For instance, hypertensive patients were prescribed enalapril, while others were on ibuprofen and co-trimoxazole (for the prevention of opportunistic infections). These medicines have the potential to contribute towards renal deterioration.^9^ Furthermore, medicines, such as paracetamol and fluconazole, which are known for their hepatotoxic effects, were found in some patient records.^9^ As illustrated in Table 3, the various medicines that were identified in patient files were recorded as follows:

**TABLE 3:** Medicines recorded with potential adverse effects.

Adverse effect – Classification	Drug	Number of patients
Renal	Aspirin 150 mg	1
	Enalapril 10 mg	2
	Hydrochlorothiazide 12.5 mg	4
	Ibuprofen 400 mg	1
Renal total	-	8
Hepatic	Alcohol	1
	Amlodipine 5 mg	3
	Atorvastatin 10 mg	4
	Azithromycin 500 mg	3
	Carvedilol	1
	Chlorpheniramine 12 mg	1
	Co-Trimoxazole 960 mg	7
	Fluconazole	1
	Lansoprazole 30 mg	1
	Omeprazole 30 mg	1
	Paracetamol 1000 mg	2
	Amitriptyline 25 mg	1

**Hepatic total**	-	**26**

Consequently, these additional medicines, as depicted in Table 3, were also taken into account because of their potential contribution to the patient’s renal and/or hepatic deterioration. As a result of the lack of information on the diagnosis of patients, current therapies were not excluded from the study.

## Discussion

In the present study, more than half of the patients were females, which confirmed the fact that women are disproportionately affected by HIV.^10^ In 2017, 26% of women in South Africa, aged between 25 and 49 years, were estimated to be living with HIV, compared to around 15% of men of the same age.^10^

According to Kim,^11^ an estimated 18.7% of the population, aged between 15 and 49 years, are HIV-positive. The results of the current study found the majority of the study participants to be between the ages of 18 and 50 years.

The age distribution of this study, which had a median age of 50 years, is in close proximity to the findings of a study conducted in the United States of America by Sebastiani,^12^ which recorded a median age of 49 years for people living with HIV and liver diseases.

The CD4 count recorded during the current study found that not all patients had their CD4 counts recorded in their files for the different 6-month intervals. This finding is also similar to that of a research study conducted by Thanawuth^13^ in Southern Thailand, which discovered that only 34% and 47% of patients received CD4 assessments within 6 and 12 months of HIV diagnosis, respectively.^13^

In this study, compliance to guidelines when prescribing ART medicines during regimen adjustments was at 21.6%, which is contrary to the findings of a study carried out in Spain by Serrano-Villar,^14^ which reported that 89.3% of patients were on regimens that complied with the guideline. The current study found a low number of renal failure patients (*n* = 12; 41.3%) who had dose regimens adjusted according to the South African national consolidated guidelines.^15^ Similar research, conducted in the United States of America, sought to establish the level and extent of compliance to guidelines concerning renal drug-dose adjustment. It was found that 43.2% of patients received at least one inappropriately dosed medication.^3^

In the present study, the quantification of liver diseases was generalised, and a total of 21.6% of patients were identified. A study conducted by Mirira^16^ found the incidence of hepatotoxicity in HIV patients to be 21% and most severe within the first 2 months of initiation. This finding was corroborated by a study conducted by Gebremiceal^17^ in Ethiopia, which found that the prevalence of hepatotoxicity in patients taking ART was 21.8%.

In this study, 75% of patients with hepatic failure did not have their regimens adjusted according to guidelines. Similarly, in a study conducted in the United States of America, Sulkowski^18^ found that severe hepatotoxicity was observed in 10.4% of patients infected with HIV. However, no significant dose adjustments were detected in hepatotoxicity incidence.

A total of 27% of patients defaulted on their treatment regimen, identified through virological failure. Similar results were reported by Peter et al.^3^ in India, where 33% of patients were non-adherent to their treatment regimen. However, a South African study carried out within a structured ART environment found patients to be more than 95% compliant for each year on ART.^19^ Results of the current study found that 27% of patients with virological failure required dose regimen adjustment. Of these regimen changes, 40% complied with the guidelines. A study conducted by Ross^20^ had similar findings, with 36.65% of patients with virological failure requiring ART regimen adjustments.

A small percentage of patients (6.1%) with HIV infection were presented with both renal and hepatic failure in the present study. These results corroborated with the results from a study undertaken by Anabire,^21^ which found 9.1% of HIV-infected patients with both renal and hepatic failure.

At the time of data collection, the National Department of Health was in the preliminary stage of phasing out the NNRTI (EFV-containing regimen). As per the guidelines, the Dolutegravir (DTG) regimen rollout at Tshepang Clinic followed an approach where patients were only migrated to DTG if they were virally suppressed (indicating adherence) among other factors. As the study collected retrospective data, the DTG regimen was not applicable nor had an effect on the data.

Antiretroviral prescribing errors and failures to comply with prescribed guidelines are not only limited to renal failures (58%) but also to hepatic (75%) and virological failures (60%), which were found in high incidence in the current research. A study conducted in the United States of America by Grissinger^22^ found that some prescribing errors included incorrect dosing frequencies and dosages (16.7%), drugs that were contraindicated for certain HIV patients (5.2%) and shortage of medicine stock (3.8%).

In this study, medicines prescribed for co-morbidities and opportunistic infections, such as enalapril and ibuprofen, which are known to cause renal toxicity, were recorded. Another study conducted by Sulkowski^18^ in the United States of America is in line with the above and found that medicines, such as aspirin, caused chronic interstitial nephritis, while medicines, such as ibuprofen, caused glomerulonephritis. Moreover, amitriptyline causes rhabdomyolysis and lastly, enalapril can lead to altered intraglomerular hemodynamics.^23^

In a study conducted by Mounzer,^24^ it was found that patients initiated on 150 mg 3TC had higher HIV ribonucleic acid (RNA), lower estimated glomerular filtration rate (eGFR) and more co-morbidities than patients initiated on 300 mg when comparing both groups. There were significant differences in the incidence of adverse diagnoses or severe laboratory abnormalities with 300 mg compared to 150 mg.

Based on the 2020 National Treatment Guidelines,^15^ tenofovir is contraindicated in patients with an eGFR of < 50 mL/min per 1.73m^2^.

The national consolidated guidelines of 2020^15^ outline that CD4 count should be measured to monitor susceptibility to opportunistic infections and eligibility for co-trimoxazole preventative therapy. Blood tests are conducted at 6 and 12 months of ART and repeated every 6 months thereafter until patients’ viral loads are recorded at below 1000 c/mL.^8^

The study site being a state-owned facility with limited resources restricted the exploration of the genetic diversity of patients and different strains of HIV. At the point of pilot data collection, the researcher discovered that the information on HIV strains and genetic information was not available on the file; therefore, it was omitted from the data collection.

## Conclusion

As treatment guidelines are fundamental to the effective management of HIV in South Africa, the findings of this study reveal a scenario that contradicts the intended purpose of these guidelines. Specifically, not all regimen adjustments were made in accordance with the established guidelines when required across all categories. The study found that for patients with hepatic, renal and/or virological failure, a non-compliance count of over 40% in each category existed.
